# The Husband’s Part

**Published:** 1890-10

**Authors:** 


### THE HUSBAND'S PART.

The home ought to be a harbor of rest; but if the wife ought to make it so for the husband, none the less ought the husband to make it so for the wife. If she should greet him with a restful presence, he should bring to her a cheerful one. The man who holds his umbrella over himself and leaves his wife to take the drippings, is a boor; but that is what not a few of us husbands do in running under shelter from all household cares and leaving our wives to take the pitiless rain of pelting perplexities.

It is said of Governor Jewell, that when he was carrying on his shoulders the burdens of a great business, and all the political anxieties of a great presidential campaign, he always brought to his home a bright face and a cheery word, and a seemingly light heart ; so that care flew out of the window when he entered the door. In this, as in all other phases of life, unselfishness is the truest and best service of self.

The man who takes best care of his wife finds in that very act the best refuge from the stinging cares of his own business. The wife ought always to feel the load lifted off her shoulders when her husband crosses the threshold in the evening. But she does not always. Sometimes it even settles down upon her shoulders heavier than before. What say you, gentlemen ?—*Christian Union.*

